# Impact of Remote Monitoring on Hospitalizations for Heart Failure: A Five-year Single-center Experience

**DOI:** 10.19102/icrm.2021.120802

**Published:** 2021-08-15

**Authors:** Jonathan Rosman, Murray Rosenbaum, Eric Berkowitz, E. Martin Kloosterman

**Affiliations:** ^1^Cardiac Arrhythmia Service, Boca Raton Regional Hospital, Delray Medical Center, Boca Raton, FL, USA; ^2^Department of Clinical Biomedical Sciences, FAU Medical School, Boca Raton, FL, USA

**Keywords:** Congestive heart failure, defibrillator, intrathoracic impedance, remote monitoring

## Abstract

The impact of a provider-driven assessment and treatment algorithm based on remote OptiVol (Medtronic, Minneapolis, MN, USA) fluid index levels on hospitalizations for congestive heart failure (CHF) remains unknown. We implemented a physician-guided screening and educational strategy for elevated OptiVol fluid index levels measured on remote implantable cardioverter-defibrillator (ICD) monitoring and assessed clinical outcomes over a five-year period. Patients with CHF and a left ventricular ejection fraction (LVEF) of 40% or less with a previously implanted ICD underwent monthly remote monitoring from January 2015 to November 2019. An OptiVol fluid index of 60 Ω-days or more triggered a protocol-based CHF screening and therapy adjustment according to clinical presentation. Among 279 patients included in the study, 228 (81%) were male and 205 (73%) had ischemic cardiomyopathy. The average LVEF was 29% (± 7.3%). A total of 6,616 monthly transmissions were reviewed over five years; of those, 575 (8.7%) were associated with elevated OptiVol fluid index levels in 178 (64%) patients, and clinical follow-up data were available in 459 of 575 (80%) cases. Following abnormal OptiVol fluid levels on remote monitoring, CHF hospitalization occurred in 10 of 459 (2.2%) patient cases. In conclusion, monthly remote monitoring of OptiVol fluid index levels with a health care provider–guided CHF screening and an educational approach to abnormal OptiVol fluid index levels were associated with a low CHF hospitalization rate. This compared favorably to prior similar studies, and randomized controlled prospective studies evaluating similar algorithms are warranted.

## Introduction

Congestive heart failure (CHF) poses a major financial burden on the health care system, particularly as it relates to the significant costs associated with recurrent cardiovascular hospitalizations.^1^ Implantable cardioverter-defibrillators (ICDs) have the capability to measure intrathoracic impedance values, which correlate with pulmonary fluid status.^2–4^ Low intrathoracic impedance values often precede clinical manifestations of CHF.^3–10^ Nevertheless, significant controversy remains regarding the impact of the utilization of ICD-derived intrathoracic impedance values on clinical outcomes, likely due to a wide variation in clinical follow-up protocols.^11–17^ In the present study, we aimed to assess the feasibility of an algorithmic approach to monthly remote monitoring of intrathoracic impedance measurements coupled with a health care provider–guided screening and education program and its impact on hospitalizations for heart failure.

## Methods

**Figure 1:**
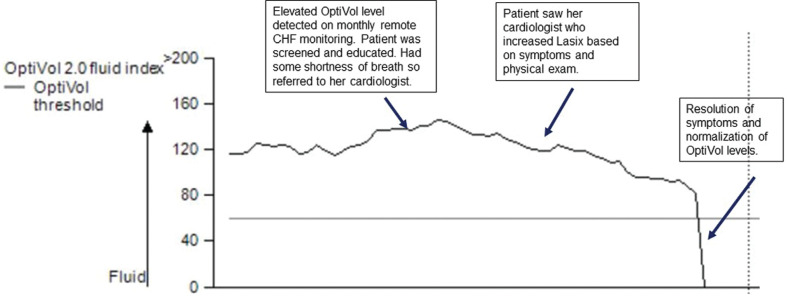
An example of a patient in the CHF remote monitoring program.

Patients with systolic CHF, a left ventricular ejection fraction (LVEF) of 40% or less, and a previously implanted ICD (Medtronic Inc., Minneapolis, MN, USA) with remote monitoring capabilities were included. Remote monitoring was performed monthly from January 2015 to November 2019 and reviewed by a cardiac electrophysiologist. Low intrathoracic impedance measurements were defined as an OptiVol (Medtronic) fluid index level of 60 Ω-days or more at the time of monthly measurement. Patients with abnormal intrathoracic impedance values were managed as follows **(Figure 1)**:

1. Patients were called by a physician assistant trained in CHF management who assessed them for signs and symptoms of CHF and provided education on diet, weight monitoring, and recognition of the signs and symptoms of CHF.
2. Patients with no reported clinical CHF manifestations were instructed to self-monitor for signs and symptoms of CHF. Patients with stable CHF symptoms were instructed to see their cardiologist if their symptoms worsened, whereas patients with new or worsening CHF signs and symptoms were referred to their cardiologist for further evaluation and management. In the latter instance, the electrophysiologist contacted the patient’s cardiologist.
3. Patients were contacted again the following month to reassess their clinical status and document whether they had been evaluated and treated by their cardiologist or had been hospitalized for heart failure. Changes to medications were also noted.

This was a retrospective chart review of de-identified patient data.

## Results

Two hundred seventy-nine patients over the five-year remote monitoring period met the criteria for analysis; of those, 59 (21%) died over the course of the study. Baseline characteristics are listed in **Table 1**. Their mean age was 78 years (± 9.1 years), and 227 patients (81%) were male. Two hundred four (73%) patients had ischemic cardiomyopathy, and the average LVEF was 29% (± 7%). One hundred and twenty-nine (46%) patients had class III CHF and 183 patients (64%) had biventricular defibrillators.

A total of 6,616 monthly transmissions were analyzed over the five years; of those, 575 (8.7%) were associated with an elevated OptiVol fluid index. Abnormal OptiVol fluid index levels were present on at least one monthly remote transmission in 178 (64%) patients. Clinical follow-up from the 575 abnormal OptiVol fluid index level transmissions was available in 459 (80%) patient cases. At the one-month follow-up for these 459 cases, 221 (48%) patients had seen their cardiologist and 90 (20%) had undergone a medication adjustment. The overall 30-day CHF hospitalization rate was 2.2% (10/459 patient cases).

The 10 hospitalizations occurred in eight patients, with one patient hospitalized on three separate occasions. Four of the patients were already hospitalized at the time of our initial phone call. Of the remaining six hospitalizations, four patients documented symptoms during our phone conversation and had seen their cardiologist prior to their hospitalization. Two patients reported no symptoms during our phone conversation but subsequently developed symptoms and were hospitalized.

## Discussion

CHF management is a major economic burden on the United States health care system with the majority of health care expenditures resulting from hospitalizations for CHF exacerbation. Specifically, the estimated cost of CHF hospitalizations in the United States was over $11 billion in 2014, and with a growing aging population, the expected yearly cost of CHF management is expected to exceed $65 billion by 2030.^1^ CHF hospitalizations are further associated with a lower quality of life and increased morbidity and mortality.^18–20^ As such, implementation of outpatient modalities and clinical algorithms that allow for early identification of CHF exacerbation and appropriate medical intervention is a crucial component in the long-term strategic approach to patients with cardiomyopathy and CHF.

We previously showed that a targeted and health care provider–guided OptiVol monitoring and intervention strategy minimized CHF hospitalizations in patients with abnormal remote intrathoracic impedance values, using a cutoff of 80 Ω-days or more for abnormal OptiVol levels.^17^ In the current study, we present 30-day CHF hospitalization rates following an abnormal OptiVol fluid index level on remote monitoring. We analyzed more than 6,500 transmissions in 279 patients over a five-year period. In contrast to our prior report, the present study defined abnormal intrathoracic impedance values as OptiVol fluid index levels of 60 Ω-days or more. All patients with abnormal intrathoracic impedance values were educated on the clinical recognition of CHF. As abnormal intrathoracic impedance values can normalize without any intervention, patients were referred to their cardiologist for further evaluation and management only when they reported clinical signs and symptoms of CHF. Primary cardiologists were then instructed to treat based on clinical and/or laboratory findings of CHF and not solely based on abnormal intrathoracic impedance levels. The remote screening protocol allowed for early clinical referral to a primary cardiology provider for in-person evaluation when needed. However, in the absence of clinical signs or symptoms of CHF, patients benefited by learning how to prevent and recognize clinical manifestations of CHF.

Prior studies have shown that patients with decreased intrathoracic impedance measurement are at a higher risk of CHF hospitalization in the ensuing weeks.^3–10^ Nevertheless, intrathoracic impedance is an early measurement of pulmonary fluid accumulation and can normalize without any intervention. It is therefore difficult to predict progression to clinical CHF and hospitalization. The Program to Access and Review Trending Information and Evaluate Correlation to Symptoms in Patients With Heart Failure (PARTNERS-HF) study analyzed 694 patients with biventricular ICDs, using an algorithmic approach including monitoring of intrathoracic impedance, low patient activity, and atrial fibrillation, and reported a 30-day hospitalization rate of 3.9%.^6^ Cowie et al. similarly used a monthly remote CHF monitoring algorithm and reported a 30-day hospitalization rate of 6.8% in their higher-risk patient group.^3^ Similarly, Triage-HF identified a high-risk group utilizing remote monitoring and reported a 30-day hospitalization rate of 6.9%.^21^ Our study findings compare favorably to these studies and indicate that the implemented direct health care provider–guided approach resulted in very low 30-day hospitalization rates. Certain differences and similarities are notable and likely explain the differences in the overall reported hospitalization rates among studies. Similar to PARTNERS-HF and Cowie et al.’s study, the present study implemented a monthly monitoring period in order to allow for early capture of abnormal impedance values in high-risk patients. As an abnormal intrathoracic impedance triggered a screening and education program rather than direct intervention, patients whose intrathoracic impedance would have normalized regardless of any intervention were not overtreated. It is possible that an even more frequent evaluation period could have prevented hospitalizations, specifically four of the 10 hospitalizations in our study that occurred prior to our phone call. Further studies evaluating shorter monitoring periods are warranted.

Our results are in contrast with prior studies that attempted to use intrathoracic impedance to reduce CHF hospitalizations.^11,12,21,22^ The Diagnostic Outcome Trial in Heart Failure (DOT-HF), Lung Impedance Monitoring in Treatment of Chronic Heart Failure (LIMIT-CHF), and the Monitoring Resynchronization Devices and Cardiac Patients (MORE-CARE) trials all analyzed the usefulness of an audible alert to detect abnormal OptiVol fluid index levels. DOT-HF saw an increase in CHF hospitalization, and LIMIT-CHF and MORE-CARE showed no benefit of an audible alert for abnormal OptiVol fluid index levels. In DOT-HF and LIMIT-CHF, the intrathoracic impedance was a main determinant in patient treatment and intervention. While our rate of cardiology visits was similar to that seen in DOT-HF, medication adjustments and hospitalizations were significantly reduced. Although 48% of our patients with decreased intrathoracic impedance measurements saw their cardiologist over the following month, only 20% of patients had a change in their CHF medication, compared to a 50% medication change in the DOT-HF trial^12^ and 100% medication change in the LIMIT-CHF trial.^11^

MORE-CARE compared remote monitoring and traditional in-office visits with an audible alert for abnormal intrathoracic impedance.^21,22^ There was no significant difference in CHF hospitalization between the two groups. In these prior studies, the audible alert may have also triggered patient treatment and intervention even in the absence of clinical signs and symptoms of CHF. In our study, cardiology visits and medication changes were a result of signs, symptoms, or a physical exam consistent with CHF, not solely based on abnormal intrathoracic impedance measurements. The ability in our study to only treat the higher-risk patients resulted in a more targeted intervention.

### Study limitations

This was a retrospective assessment of a single group’s clinical practice and not necessarily applicable to all practices. Clinical follow-up was not available in 20% of patient cases. While this follow-up is similar to prior studies,^10^ we cannot exclude potential exclusion bias. We chose a threshold for OptiVol fluid index level greater than 60 Ω-days, which allowed for increased sensitivity but also decreased specificity.

## Conclusions

We present a five-year single-center study utilizing monthly remote OptiVol fluid index levels combined with a health care provider–guided screening and educational and early referral approach to reduce CHF hospitalizations. The 30-day hospitalization rate following monthly remote monitoring of abnormal OptiVol fluid index levels was low (2.2%) and compared favorably to prior studies. Randomized controlled prospective studies evaluating similar algorithms are warranted.

## Figures and Tables

**Table 1:** Baseline Characteristics of Patients with Abnormal OptiVol Levels

Variable	n = 279
Age, years	82 ± 8
Female, n (%)	51 (18.2)
Heart failure etiology, n (%)	
Ischemic	205 (73.2)
Nonischemic	75 (26.8)
Left ventricular ejection fraction, n (%)	29.0 ± 7.3
BMI, kg/m^2^	26.7 ± 5.5
Hypertension, n (%)	111 (39.8)
Diabetes, n (%)	52 (18.6)
Atrial fibrillation, n (%)	126 (45.2)
NYHA functional class, n (%)	
I	4 (1.4)
II	140 (50.2)
III	129 (46.2)
IV	6 (2.2)
ACE inhibitor/angiotensin receptor blocker, n (%)	168 (60)
β-blocker, n (%)	235 (84)
Diuretic, n (%)	172 (62)

ACE: angiotensin-converting enzyme; BMI: body mass index; NYHA: New York Heart Association.

## References

[1] Jackson SL, Tong X, King RJ (2018). National burden of heart failure events in the United States, 2006 to 2014. Circ Heart Fail.

[2] Abraham WT, Compton S, Haas G (2011). Intrathoracic impedance vs daily weight monitoring for predicting worsening heart failure events: results of the Fluid Accumulation Status Trial (FAST). Congest Heart Fail.

[3] Cowie MR, Sarkar S, Koehler J (2013). Development and validation of an integrated diagnostic algorithm derived from parameters monitored in implantable devices for identifying patients at risk for heart failure hospitalization in an ambulatory setting. Eur Heart J.

[4] Yu CM, Wang L, Chau E (2005). Intrathoracic impedance monitoring in patients with heart failure correlation with fluid status and feasibility of early warning preceding hospitalization. Circulation.

[5] Small RS, Wickemeyer W, Germany R (2009). Changes in intrathoracic impedance are associated with subsequent risk of hospitalizations for acute decompensated heart failure: clinical utility of implanted device monitoring without a patient alert. J Card Fail.

[6] Whellan DJ, Ousdigian KT, Al-Khatib SM (2010). Combined heart failure device diagnostics identify patients at higher risk of subsequent heart failure hospitalizations: results from PARTNERS HF (Program to Access and Review Trending Information and Evaluate Correlation to Symptoms in Patients With Heart Failure) study. J Am Coll Cardiol.

[7] Sarkar S, Hettrick DA, Koehler J (2011). Improved algorithm to detect fluid accumulation via intrathoracic impedance monitoring in heart failure patients with implantable devices. J Card Fail.

[8] Binkley PF, Porterfield JG, Porterfield LM (2012). Feasibility of using multivector impedance to monitor pulmonary congestion in heart failure patients. J Interv Card Electrophysiol.

[9] Ahmed FZ, Taylor JK, Green C (2020). Triage-HF Plus: a novel device-based remote monitoring pathway to identify worsening heart failure. ESC Heart Fail.

[10] Virani SA, Sharma V, McCann M (2018). Prospective evaluation of integrated device diagnostics for heart failure management: results of the TRIAGE-HF study. ESC Heart Fail.

[11] Domenichini G, Rahneva T, Diab IG (2016). The Lung Impedance Monitoring in Treatment of Chronic Heart Failure (the LIMIT-CHF study). Europace.

[12] Van Veldhuisen DJ, Braunschweig F, Conraads V (2011). Intrathoracic impedance monitoring, audible patient alerts, and outcome in patients with heart failure. Circulation.

[13] Van Veldhuisen DJ, Maass AH (2012). Telemonitoring of outpatients with heart failure: a search for the holy grail?. Circulation.

[14] Morgan JM, Kitt S, Gill J (2017). Remote management of heart failure using implantable electronic devices. Eur Heart J.

[15] Böhm M, Drexler H, Oswald H (2016). Fluid status telemedicine alerts for heart failure: a randomized controlled trial. Eur Heart J.

[16] Hawkins NM, Virani SA, Sperrin M (2016). Predicting heart failure decompensation using cardiac implantable electronic devices: a review of practices and challenges. Eur J Heart Fail.

[17] Rosman J, Rosenbaum M, Kloosterman EM (2016). A Patient-centered educational approach to intrathoracic impedance remote monitoring can reduce hospitalizations. Innov Card Rhythm Manag.

[18] Yancy CW, Jessup M, Bozkurt B (2013). ACCF/AHA guideline for the management of heart failure: a report of the American College of Cardiology Foundation/American Heart Association Task Force on Practice Guidelines. J Am Coll Cardiol.

[19] Iqbal J, Francis L, Reid J (2010). Quality of life in patients with chronic heart failure and their careers: a 3-year follow-up study assessing hospitalization and mortality. Eur J Heart Fail.

[20] O’Connor, Christopher M, Whellan DJ (2012). Factors related to morbidity and mortality in patients with chronic heart failure with systolic dysfunction: the HF-ACTION predictive risk score model. Circ Heart Fail.

[21] Burri H, Quesada A, Ricci RP (2010). The Monitoring Resynchronization Devices and CARdiac patiEnts (MORE-CARE) study: rationale and design. Am Heart J.

[22] Boriani G, Da Costa, Quesada A (2017). Effects of remote monitoring on clinical outcomes and use of healthcare resources in heart failure patients with biventricular defibrillators: results of the MORE-CARE multicentre randomized controlled trial. Eur J Heart Fail.

